# The Imprint of Exposome on the Development of Atopic Dermatitis across the Lifespan: A Narrative Review

**DOI:** 10.3390/jcm12062180

**Published:** 2023-03-11

**Authors:** Katerina Grafanaki, Angelina Bania, Eleni G. Kaliatsi, Eleftheria Vryzaki, Yiannis Vasilopoulos, Sophia Georgiou

**Affiliations:** 1 Department of Dermatology, School of Medicine, University of Patras, 26504 Patras, Greece; 2 Faculty of Medicine, School of Health Sciences, University of Patras, 26504 Rion, Greece; 3 Department of Biochemistry, School of Medicine, University of Patras, 26504 Patras, Greece; 4 Laboratory of Genetics, Section of Genetics, Cell Biology and Development, Department of Biology, University of Patras, 26504 Patras, Greece

**Keywords:** exposome, atopic dermatitis, age, skin of color, COVID-19, war

## Abstract

Atopic dermatitis (AD) is a chronic inflammatory skin condition that affects more than 200 million people worldwide, including up to 20% of children and 10% of the adult population. Although AD appears frequently in childhood and often continues into adulthood, about 1 in 4 adults develop the adult-onset disease. The prenatal period, early childhood, and adolescence are considered critical timepoints for the development of AD when the exposome results in long-lasting effects on the immune system. The exposome can be defined as the measure of all the exposures of an individual during their lifetime and how these exposures relate to well-being. While genetic factors could partially explain AD onset, multiple external environmental exposures (external exposome) in early life are implicated and are equally important for understanding AD manifestation. In this review, we describe the conceptual framework of the exposome and its relevance to AD from conception and across the lifespan. Through a spatiotemporal lens that focuses on the multi-level phenotyping of the environment, we highlight a framework that embraces the dynamic complex nature of exposome and recognizes the influence of additive and interactive environmental exposures. Moreover, we highlight the need to understand the developmental origins of AD from an age-related perspective when studying the effects of the exposome on AD, shifting the research paradigm away from the per se categorized exposome factors and beyond clinical contexts to explore the trajectory of age-related exposome risks and hence future preventive interventions.

## 1. Introduction

Atopic dermatitis (AD) or atopic eczema is a common inflammatory skin disease with early onset which affects 200 million people worldwide, including up to 20% of children and 10% of adults, but the prevalence of the disease varies greatly throughout the world. In general, AD prevalence is driven by a complex relationship between environmental, genetic predispositions, and immunologic factors. An awareness of environmental diversity is crucial in determining disease phenotypes. More specifically, AD is recognized as a heterogeneous disease with multifactorial etiology and complex pathophysiology involving the immune system and epidermal barrier dysfunction, which are influenced by genetic, epigenetic and environmental factors [1,2]. Genes encoding skin barrier proteins, such as filaggrin (FLG), have been shown to play a role in the inheritance of AD. FLG is synthesized from profilaggrin, which is then transformed into FLG monomers which in turn interact with intermediate filaments in the stratum corneum (SC), causing them to aggregate into dense parallel arrays of macrofilaments. This promotes cellular compaction and keratin crosslinking in the SC, which forms a highly insoluble matrix that acts as a protective barrier. Filaggrin deregulation and loss-of-function (LOF) variants leading to abnormal FLG production result in skin barrier disruption in individuals carrying FLG LOF variants and are characterized by dry, cracked and infection-prone skin [3]. Dysfunction of the skin barrier is a cardinal clinical sign of AD as this facilitates allergen penetration and immune dysfunction and has been associated with the etiopathogenesis of the AD itch-scratch cycle. This complex pathophysiology translates into heterogeneous clinical phenotypes with differences depending on the age of onset, severity, sensitization profiles, disease persistence, presence of comorbid atopic and nonatopic conditions and longitudinal trajectories of disease progression [4].

The exposome can be defined as the measure of all the exposures of an individual during their lifetime and how these exposures relate to well-being. The external environmental exposures (external exposome) to which an individual is exposed before and after conception and their consequences at the organ and cellular level (internal exposome) are examined to explain the onset, development, and exacerbations of allergic diseases such as AD. Humidity, pollution, ultraviolet ray exposure, average time spent indoors, lifestyle and allergen exposure vary widely throughout different regions and may aggravate AD. Climate change, urbanization and loss of biodiversity are affecting the sources, emissions and concentrations of major airborne allergens and air pollutants and are among the most critical health and quality of life challenges for the increasing number of allergic patients both today and in future decades [5]. As such, the exposome has emerged as a key factor in the development of AD during the lifespan and it is now studied in correlation to the known genetic or biochemical variations that contribute to AD onset.

Globally, the prevalence of AD increases with socioeconomic status and is usually higher in high-income countries, with variations between and within countries. AD may be underdiagnosed in patients with skin of color due to lack of erythema, and therefore, black children are six times more at risk for increased disease severity [6]. African American children and infants of color have a higher prevalence than white American children and infants (37.0% for Black, 25.8% for Asian, 24.1% for Hispanic, 23.0% for multiracial and 17.9% for White). Studies have demonstrated a nearly twofold higher prevalence of AD in black-Caribbean children compared to whites [7]. In addition, the increased prevalence of AD in black patients may extend into old age; furthermore, the socioeconomic environment appears to play a role, as differences in economic burden may reflect in the disease burden [8,9].

**Figure 1:**
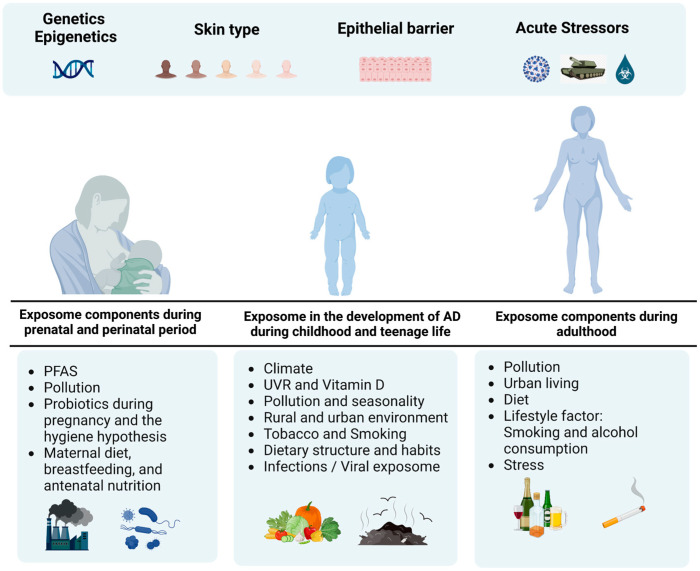
**Exposome on the development of atopic dermatitis across the lifespan.** Genetic, epigenetic, skin type and epithelial dysfunction only partially explain AD onset. The exposome including pollution, climate, rural and urban environment, lifestyle factors and acute stress differentially affects the trajectory of AD starting from conception, during pregnancy, childhood, adulthood and across the lifespan.

Notably, AD prevalence varies between urban and rural populations. The presence of patients in rural areas with limited healthcare access likely results in an underrepresentation of AD prevalence estimations in these areas. Changes in the composition of the gut and skin microbiome due to environmental or lifestyle factors are key mediators of allergic diseases (Figure 1). A better understanding of the impact of external exposome on the development of AD is crucial to encourage patients, health professionals and policymakers to take action to mitigate the impact of and adapt to environmental changes [10]. 

Previous reviews have attempted to summarize the multitude of factors contributing to AD prevalence and progression, which illustrates the necessity of understanding the exposome to manage AD. However, the exposure to these factors varies not only from person to person but also over the course of a patient’s lifetime. In this review, we aim to provide an overview of the AD exposome as an ever-changing variable over the human lifespan. By highlighting patient uniqueness and the temporal variability of their environment in the context of the natural history of AD, insights to guide better population-scale as well as personalized interventions could emerge as an important tool for effective and personalized treatment.

## 2. The Role of Exposome Components during Prenatal and Perinatal Period

### 2.1. Perfluoroalkyl and Polyfluoroalkyl Substances (PFASs)

PFASs, widely known as “forever chemicals”, are a large group of compounds used for non-stick or stain-resistant surfaces of many products, including several household items. They are widely used in industrial products, i.e., lubricants, surfactants, fire-fighting foams and in everyday products, such as non-stick cookware, greaseproof paper, food packaging, carpets, furniture, waxes, paints, clothing, and personal care products such as shampoo, eye-makeup, nail varnish, and dental floss (Figure 1). Prenatal exposure to PFAS, such as perfluorooctane sulfonate (PFOS), perfluorooctanoic acid (PFOA), perfluorohexane sulfonate (PFHxS), perfluorononanoic acid (PFNA), and perfluorodecanoic acid (PFDA), has been detected in 90% or more of pregnant women in the US, Europe, and Asia and are associated with childhood AD in girls during the first 2 years [11]. In addition, prenatal exposure to polychlorinated biphenyls (PCBs) increased the risks for asthma and eczema in offspring [12]. Prenatal exposure to PFAS occurs through placental transfer in utero and postnatal exposure through breastfeeding and has been associated with a higher risk of early AD in children under 5 years of age [13,14]. Studies have correlated prenatal PFOA and PFOS exposures with elevated cord blood IgE levels, especially in boys, and it has been hypothesized that PFAS might augment hypersensitivity to allergens [15]. Moreover, in utero exposure to PFOA has been associated with a higher risk of AD development as early as the age of 2 years old, with children carrying GSTT1-null or GSTM1-null genotypes that affect the glutathione S-transferase (GST) activity which is essential in chemical detoxification. GSTM1-null and GSTP1 Ile/Ile genotypes are also associated with increased risk of AD in children with prenatal smoke exposure [16]. Finally, in the home environment, exposure to PFAS places infants and young children at increased risk of exposure due to their exploring and hand-to-mouth behavior. 

### 2.2. Pollution

On the other hand, air pollution which is the contamination of the indoor or outdoor environment by any chemical, physical or biological agent that modifies the natural characteristics of the atmosphere can trigger AD onset in childhood. Domestic combustion appliances, motor vehicles, industrial installations and forest fires are common sources of pollutants. Pollutants of major public health concern are grouped into air pollutants (e.g., sulfur dioxide, nitrogen oxide, carbon monoxide, ozone and volatile organic compounds), persistent organic pollutants (e.g., dioxins), heavy metals (e.g., cadmium, lead, mercury) and particulate matter (PM). Exposure to lead in late pregnancy increases the risk of AD in boys at 6 months of age. Prenatal exposure to inorganic arsenic and co-exposure to inorganic arsenic and cadmium were associated with a higher risk of AD in young children [17]. Mono-benzyl phthalate (MBzP) increased the risk of developing eczema in early childhood [18]. Additionally, the combination of prenatal exposure to bisphenol A (BPA) and phthalates could be associated with AD in 6-month-old infants [19]. 

According to the US Environmental and Protection Agency (EPA), PM is classified according to particle size; hence, PM0.1 (ultrafine particles, ≤0.1 μm), PM2.5 (fine particles, ≤2.5 μm), PM10 (coarse particles, ≤10 μm) [20]. PM causes skin barrier dysfunction and the formation of reactive oxygen species, leading to induced oxidative stress, possibly epigenetic changes, and skin inflammation via direct and indirect mechanisms [20]. Of interest, is the association between maternal exposure to traffic-related pollution and the prevalence of AD in offspring. 

Air pollution and the incidence of AD in East Asia were investigated in birth cohort studies, in which exposure to typical traffic-related air pollution from exhaust fumes during pregnancy resulted in increased rates of AD in children, whereas higher concentrations of particulate (PM10, PM2.5) and gaseous (NO_2_, VOC, O_3_, SO_2_) air pollutants increased the intensity of symptoms of existing AD. The effects of prenatal exposure to PM over three trimesters on skin barrier dysfunction were studied. Prenatal exposure to PM in the first trimester and skin barrier dysfunction were positively associated with AD in offspring, early-onset, and greater severity at three years of age. Notably, higher PM2.5 in the first trimester of pregnancy, higher maternal prenatal stress and male sex were associated with AD at age 1. In addition, a significant association between PM2.5 exposure among younger AD individuals aged between 2–30 years old, but not for PM10 exposure has been described. With their smaller size and higher number of metal components, PM2.5 can easily penetrate deep into skin cells and therefore may cause a higher risk of AD than PM10. Interestingly, high levels of PM2.5 during the first trimester and low cord blood vitamin D influenced the early onset of persistent AD, with the most sensitive period being 6 to 7 weeks of gestation mediated by placental DNA methylation [21]. Furthermore, increased maternal exposure to fine PM synergistically interacts with postnatal environmental tobacco smoke inhalation on the development of infantile eczema. Gestational and prenatal exposure to cigarette smoke were both correlated with the development of adult-onset AD. Smoking may affect the immune processes of babies born to smoking mothers, which may have a reduced innate immune response.

### 2.3. Probiotics during Pregnancy and the Hygiene Hypothesis

Supplementation with probiotics by pregnant women during prenatal and postnatal lactation and postnatal fetuses can efficiently reduce the risk of AD in children, while the effect of multi-strain probiotic mixtures is superior to single-strain formulations [22,23]. In an 11-year, double-blind, placebo-controlled study, pregnant women in the experimental group continued to receive probiotics from 35 weeks gestation to 6 months postpartum, and infants received oral probiotics from birth till the age of 2 years. The rate of AD in the probiotic group was significantly lower than that in the placebo group. In an observational clinical trial, a multi-strain probiotic was administered orally to pregnant women from 4 weeks before delivery to 4 weeks after delivery. The secretion of sIgA in the infant’s feces suggested that oral probiotics during pregnancy and delivery contribute to the improvement of intestinal barrier function during the neonatal period [24]. 

Disorders of the intestinal microbiota are closely related to the onset and development of AD. Maternal diet, specifically a diet rich in fish, has protective effects on the fetus, due to the immunological effects of long-chain polyunsaturated fatty acids and their contribution to the homeostasis of cell membranes. In addition, alcohol consumption during pregnancy was associated with a significant and dose-dependent increased risk of AD in early infancy [25].

The hygiene hypothesis is further refined to the “old friends’ hypothesis”, implying that increases in allergies reflect a lack of exposure to beneficial microbiota, which have co-evolved with humans. These include the largely non-harmful commensal microbes acquired from the skin and gut of other humans, as well as micro-organisms such as helminths, Helicobacter pylori, and hepatitis A virus that can persist for life and need to be tolerated [26].

A meta-analysis of the role of probiotics in the prevention and treatment of AD in children shows that oral probiotics given to pregnant women and newborns can effectively reduce the prevalence of AD in children. Children with AD over 1 year of age responded better to oral probiotics, with better efficacy in children with moderate to severe AD than mild AD. In addition, both single and multi-strain preparations, especially the probiotic mixture containing lactobacillus and bifidobacterial, have a therapeutic effect with a superiority of the multi-strain preparation. Lactobacillus sp., such as *Lactobacillus acidophilus* alone or *Lactobacillus acidophilus* mixed preparations with other strains of probiotics can improve clinical symptoms in children with AD. Moreover, combinatorial use of *Lactobacillus casei* and *Lactobacillus salivarius* can reduce IgE levels. Oral and topical probiotics can regulate the distribution of local microbiota and improve the body’s immune response, thus proving to be a promising method of prevention and adjuvant treatment of AD in children [27].

### 2.4. Maternal Diet and Antenatal Nutrition

Maternal diet and antenatal nutrition could affect fetal development by altering fetal programming, which may in turn alter immune response and atopy. Nutrition during infancy and childhood is very important for the development of AD, and breastfeeding for the first 6 months is considered to be effective in preventing the development of atopic diseases (Figure 1). Moreover, breastfeeding for the first 4 months reduces the risk of eczema in the first 4 years. Additionally, feeding infants with extensively hydrolyzed formula (eHF) in the first 4–6 months, avoiding cow’s milk and dairy products, and starting solid foods after 4 months have been shown to prevent the development of AD [28].

Inadequate vitamin E intake during pregnancy was associated with a higher risk of AD in two-year-old children [29]. However, reduced risk in offspring was associated with maternal intake of beta-carotene, vitamin E, zinc, calcium, magnesium, and copper during pregnancy. While intake of allergenic foods and foods rich in n-6 polyunsaturated fatty acids during pregnancy may increase the risk of AD in offspring, foods rich in n-3 polyunsaturated fatty acids, fruits, vegetables, and prebiotics may decrease it [30]. Interestingly, an unhealthier diet—which is evaluated as a low score between Prudent Dietary Pattern (PDP) score and Western Dietary Pattern (WDP) score ((PDP-WDP) score)—during pregnancy was associated with a lower risk of atopic dermatitis and a higher risk of infections [31]. Moreover, maternal infection during gestation and exposure to antibiotics in early life was identified as a risk factor for the development of AD [32,33].

## 3. Exposome in the Development of AD during Childhood and Teenage Life

### 3.1. Climate, UVR and Vitamin D

AD exacerbates seasonally and in response to climate changes, including UV exposure, humidity, temperature, precipitation, and indoor heating. Combined high UV exposure and temperature appear to have protective effects specific to AD, in contrast to the combination of high humidity and precipitation (Figure 1). In early childhood, direct exposure to UV radiation is beneficial for reducing the risk of eczema, whereas high latitude and thus reduced exposure to UV radiation is associated with an increased risk [34,35]. In the meantime UV radiation stimulates vitamin D production, while its deficiency has been associated with increased incidence and severity of AD symptoms. Vitamin D3 supplementation significantly improves clinical symptoms in patients with AD, especially in those with winter-related AD [36]. Finally, there is an inverse association between AD severity and serum vitamin D3 levels, whereas low maternal fish consumption and reduced VD3 intake during pregnancy increase the incidence of AD in offspring [37].

Of note, Hispanic and black children are more prone to persistent AD than white children, with black children being more at-risk for incident AD in the first place. In a large study on children residing in urban US areas, black children have the highest prevalence and severity of AD among the examined ethnic groups, as well as the smallest chance for full disease control, while AD onset occurs later in life for Hispanic children [38]. Black children are also exposed to more known risk factors, including social risk factors, such as living in older or rental houses or between two homes, tobacco exposure, lower income, and lower parental educational level [39]. The latter two also affect Hispanic children. It is known that people with darker skin types have lower 25(OH)-vitamin D levels and low maternal 25(OH)D during pregnancy increases the offspring’s AD risk. Compared with non-Hispanic whites, AD incidence and persistence are higher among certain non-white racial subgroups. In a longitudinal cohort including black children with AD aged 0 to 2 years, all analyses were stratified by race. It appeared that despite lower vitamin D levels in black participants, allergic sensitization load was associated with FLG expression in the skin without lesions in non-black children, but not in black children with low vitamin D levels [40,41]. Further research is warranted to identify environmental, socioeconomic, and genetic factors that may be responsible for the observed differences.

### 3.2. Pollution and Seasonality

As children grow older and are exposed to air pollution, there is a higher prevalence or recurrence of AD in areas with high concentrations of PM, benzene, carbon monoxide, sulfur dioxide and nitrogen oxides, with a slight preference for girls over boys. A study examining a large prospective birth cohort showed a significant positive correlation between NO_2_ exposure and AD at 6 years. Moreover, a study of pediatric AD patients living in Korean urban industrial areas for two 6-month periods, showed a positive correlation between exposure to PM and exacerbation of AD, with a longitudinal association between elevated PM levels and an increase in reported AD symptoms [42,43]. 

Apart from climatic factors per se, other factors such as seasonal climate variations, fluctuations in tropospheric ozone levels and air pollutants that have seasonal trends and vary with climate, contribute to the onset of childhood AD. Interestingly, among children, there is a significant seasonal variation, with children showing symptoms mainly in late winter (“winter type”) and children showing exacerbation of symptoms in the summer, especially when exposed to grass pollen (“summer type”) [44]. 

Higher odds of eczema were found in areas with a warm, humid, and rainy climate and high levels of SO_2_, SO_3_, OC and PM2.5, while lower prevalence was found in areas with a warm and sunny climate along with high PM10 and high ozone levels, as well as in areas with high levels of NO_2_, NO_3_ and PM2.5. In contrast, CO was inversely associated with eczema severity. Higher levels of lead, zinc, nickel, vanadium and arsenic in PM2.5 were associated with increased eczema prevalence, in contrast to copper, potassium and cadmium which were inversely associated with eczema [45].

A study in children aged 0–17 years showed that in the USA, states with higher average annual NO_2_, SO_2_ and SO_3_ were associated with a higher prevalence of eczema, especially in the cold and warm months. In addition, higher levels of tropospheric ozone in the warm months, CO and PM2.5 in the cool months, and NO_3_, OC and PM10 in the cool and warm months, were associated with lower odds of eczema. Among countries, the amount of UVR exposure and AD prevalence varies between countries and depends on both latitude and stratospheric ozone concentration. As for other climatic factors, humidity, high temperatures and low precipitation are inversely correlated with AD. A possible underlying mechanism for UV radiation involvement in AD could be through the reduction in inflammation by cis-uronic acid and modulation of the skin and gut microbiome [20,45,46]. 

From a different scope, cold outdoor temperatures and exposure to shampoo were associated with eczema aggravation. Currently, it remains unclear whether parabens, which are included in many daily consumer products such as cosmetics, shampoos and personal care products as preservative antimicrobial agents, induce or aggravate it [47,48]. However, a population study suggested an increased association between children aged 0–3 years exposed to parabens [49]. In addition, water hardness is associated with eczema in genetically predisposed infants, especially in carriers of the FLG mutation. Swimming in chlorinated pools was associated with disease worsening and comprises one of the ‘irritant factors’ currently mentioned in ‘eczema school’ programs [50].

### 3.3. Rural and Urban Environment

Living on a farm or in a rural environment in childhood had a protective effect on sensitization even in middle age, but these factors did not protect from new-onset sensitization in adults [51]. It also increases exposure to a wide variety of environmental and pet allergens at an early age, which according to the hygiene hypothesis, reduces the risk of sensitization and development of atopy later in life. In contrast, it increases the risk of allergic disease later in childhood, despite the protective advantage from early life [52,53]. Exposure to a high allergen load may trigger atopy in people who are genetically susceptible. In a cohort of rural Senegalese children and teenagers under the age of 15, contact with cows significantly increased the odds of AD, while by contrast, the presence of a cat in the house showed a protective effect [54]. However, contact with pets, but not cats, has a protective effect mainly in younger ages with a favorable effect of exposure to dogs. Concerning cats, birth cohort studies showed a significant interaction between cat ownership at birth and mutations in FLG (R501X, 2282del4) on the development of early-onset AD [55]. However, a recent meta-analysis could not confirm this hypothesis, implying the complexity of gene-environment interactions in AD pathogenesis [56,57]. 

Interestingly, both physician-diagnosed and self-reported atopic eczema were rare in Russian Karelia (rural), as compared to Finnish Karelia (urban). An indication of higher microbial exposure in Russian Karelia is that skin and nasal microbiota were found to be significantly more diverse than in Finnish Karelia. In the Russian population of Karelia, contact with nature and a higher prevalence of Acinetobacter colonization was associated with protection from atopic dermatitis, alongside other allergic diseases [58,59]. On the other hand, living in a home with dampness and mold increases the risk for AD. It is important to highlight the significance of urban planning towards a green environment to improve maternal and child health following the associations of residential greenery with eczema in infants and pregnancy which appears to be the critical exposure window [60].

A much higher (25.6% vs. 2.0%) prevalence of eczema was observed in children aged 1 to 3 years in urban areas of South Africa compared to rural areas. Lower exposure to house dust endotoxin in urban and rural households was associated with a higher prevalence of AD, and significant differences in the composition of the house dust microbiota were observed between children with AD and healthy controls [61]. The detection of specific cat antigens in rural households was significantly lower in the AD cohort [41]. A reduced risk of AD was associated with urban children who consumed unpasteurized or fermented milk as well with frequent treatment with anthelmintic drugs [62]. The use of paraffin or kerosene as a means of heating reduced the odds of AD for all households. Overall significant differences in nutrition and cooking habits, contact with nature and animals, increased IL-6 and TNF-a, especially in patients from rural areas [63].

### 3.4. Tobacco and Smoking

Cigarette smoke is now an important independent risk factor in children aged 6–13 years, especially when there is a history of maternal smoking during pregnancy and infancy (Figure 1) [43]. When the disease occurs in childhood, it raises the hypothesis that a history of passive smoking becomes relevant only later in life [64]. It appears that allergic sensitization occurs over time, while tobacco smoke contributes to the destruction of the skin barrier and increased transdermal TEWL water loss, allowing contact with allergens and pathogens [65]. The worldwide prevalence of AD has been associated with active smoking [66].

### 3.5. Dietary Structure and Habits

Studies have tried to identify associations between dietary structure, nutrients, and AD. More specifically, protein consumption and especially protein derived from cereal, nuts, fish and seafood, as well as vegetables and vitamins A and E are linked with decreased AD in adolescents aged 13–14 years (Figure 1) [67]. Studies conducted on children and adults have found an inverse relationship between serum vitamin C and E levels with AD, and supplementation of vitamin E reduced AD symptoms [68,69]. The opposite, however, occurs with carbohydrates and fat, especially saturated fat, although olive oil demonstrated a negative association with eczema. In the meantime, adherence to a Mediterranean diet and consumption of fermented milk products might be protective factors for AD. In addition, specific food consumption, besides food allergies, may aggravate AD [70]. Although frequent consumption of fast foods, energy drinks, and convenience food has been related to the recently diagnosed AD in adolescents, extensive studies are needed to determine causality. Global epidemiological studies of children have shown the association of higher parental socioeconomic status with childhood allergic sensitization, likely due to a reduction in allergen exposure during immune system development by living in a healthier environment [71]. 

### 3.6. Infections/Viral Exposome

With increased hygiene and lack of exposure to bacteria, viruses and parasites, the immune system is not stimulated to develop immune responses. However, this hypothesis fails to explain the increase in allergy even in areas lacking basic hygiene services and the lack of reduction in atopic disease in those exposed to childhood viral diseases [53]. 

The composition of bacterial communities at disease preference sites was dramatically different in AD patients compared with controls. Microbial diversity during AD flares depended on the presence or absence of recent AD treatments, with even intermittent treatment being associated with greater bacterial diversity than the absence of recent treatment. In AD, the proportion of *S. aureus* was greater during disease flares than at baseline or after treatment and was associated with worsening disease severity. The S. epidermidis skin commensal also increased significantly during flare-ups. Increases in Streptococcus, Propionibacterium and Corynebacterium species were observed after treatment. Furthermore, the flexures and neck show site-specific microbial colonization. the flexures and neck. In lesions, the flexures have lower alpha diversity and a high abundance of *S. aureus*, while the neck has a high abundance of Malassezia species. [72,73,74].

The composition of the gut microbiome, although associated with allergic sensitization, does not affect the risk of atopic dermatitis in children up to 6 years of age [75]. The gut microbiome composition in young people shows a reduced presence of Bifidobacterium in AD; helminths play no role in AD in children, as they do not suffer the additional risk of eczema from anti-helminthic treatment, unlike patients with childhood AD [76].

## 4. The Exposome Effect on AD of Adulthood

### 4.1. Pollution

At least 1 in 4 cases of AD are adult-onset, with significant heterogeneity in the age of onset (Figure 1). Some adults may have the disease from infancy or school age, namely childhood-onset AD that persists into adulthood. While others may report onset in adolescence or adulthood, known as adult-onset or recurrent AD [77]. 

Throughout life, PM exposure exacerbates AD. Air pollutants cause dermal oxidative stress and have been shown to harm the integrity of the skin barrier by altering TEWL, inflammatory cascade, stratum corneum pH and the microbiome. Various oxidative stress markers in the stratum corneum of AD biopsies have shown a correlation with AD severity. Therefore, supporting the hypothesis that environmentally generated ROS may induce oxidative protein damage in the stratum corneum, leading to the disruption of barrier function and exacerbation of AD [78]. There is a perception that air pollution disproportionately affects adult AD compared to pediatric AD.

### 4.2. Urban Living

Urban living often increases exposure to environmental irritants, such as NO2 which contributes to the formation of trophospheric ozone and greenhouse gases. Short-term exposure to NO2 or VOC caused significantly increased TEWL in both healthy individuals and those with AD [79,80,81]. Interestingly, exposure to diesel exhaust and proximity to heavy traffic leads to increased IgE production, and has been shown to be an environmental risk factor associated with AD [48,82,83].

Polycyclic aromatic hydrocarbons (PAHs) fluorene and phenanthrene are potentially associated with the pathogenesis of AD in adults [84]. It is hypothesized that PAHs may exert biological effects through the binding of aryl hydrocarbon receptor (AhR) which is suggested to be overexpressed in AD patients [85]. Various organic components of pollutants interact with the AhR in keratinocytes to induce epidermal hyperstimulation through the induction of the neurotrophic factor artemin that causes nerve growth hypersensitivity pruritus and AD pathophysiology [86]. Dry environmental conditions can markedly enhance epidermal structure and function. Low humidity and low temperatures decrease skin barrier function and increase susceptibility towards mechanical stress, while the skin also becomes more reactive towards skin irritants and allergens as pro-inflammatory cytokines and cortisol are released by keratinocytes, and the number of dermal mast cells increases. Cold and dry weather appears to increase the prevalence and risk of flares in patients with atopic dermatitis [87]. However, cold alone for short periods of time did not affect TEWL or skin irritation. TEWL is greater in black skin compared with white skin because the former is characterized by a lower ceramide/cholesterol ratio loss, both contributing to skin dryness [88]. In US and Italy, increased hospital admissions occur during winter due to low temperature, humidity and UVR exposure, as well as increased indoor heating. Whereas admissions increase in the south during summer due to the high temperature and humidity, which induces increased body temperature, sweating and itching [89]. A Nigerian prospective study reported exacerbation of symptoms with hot and humid weather with tropical-black AD patients visiting the clinic in the dry season, (higher temperature and UV index and lower precipitation. Exposure to tropical meteorological variables may affect the occurrence of AD [90]. 

### 4.3. Diet

As with childhood AD, data from the 2010 NHIS suggest that increased household income, higher household education level, and households with more individuals are significantly associated with an increased prevalence of AD in adults [91].

Although some studies investigating the effects of dietary fatty acids on AD in adults have indicated that low n-3 intake is inversely correlated with AD in women, other studies have found no association between n-3 intake and AD, and other clinical studies reported that n-3 supplementation in adults did not show any benefit over placebo in AD. Oxidative stress has been shown to induce AD by increasing the pro-inflammatory response. An anti-inflammatory diet includes oily fish (high in n-3), fruit, vegetables, seeds and probiotics and limit meat, whole grains, sugar, and flour. Meantime, ‘free radical’ foods, with antioxidant qualities, include berries, cherries, citrus fruit, prunes, olives, and green tea [92,93,94].

Certain foods and dietary patterns can trigger acute changes that lead to visible skin effects. AD has increased globally across all age groups, and this increase has also been associated with the westernization of dietary patterns and increased consumption of processed food. Processed foods and some food additives such as monosodium glutamate (a popular flavor enhancer) could act as pseudo-allergens and increase the occurrence and severity of AD [95]. On the other hand, fermented food intake is associated with a reduced likelihood of atopic dermatitis in a Korean adult population [96]. 

### 4.4. Lifestyle Factors—Smoking and Alcohol Consumption

Although, there is no consistent evidence that drinking can cause eczema or a flare-up, adults with AD had higher rates of cigarette smoking and greater odds of ever drinking 12 or more alcoholic beverages in low or high quantities. Current heavier drinking was associated with eczema in all racial groups compared with a lifetime abstaining from or current light drinking. Interestingly, eczema was associated with higher odds of ever drinking 12 or more alcoholic beverages in whites (aOR, 1.15; 95% CI, 1.14–1.15), blacks (aOR, 1.46; 95% CI, 1.46–1.47), and American Indians (aOR, 5.92; 95% CI, 5.83–6.01) but not in Asian-Americans (aOR, 1.00; 95% CI, 0.99–1.00) [97].

The relationship between active and passive smoking and adult AD remains controversial. Early and current cigarette smoking, as well as exposure to environmental cigarette smoking during childhood, have both been associated with a higher incidence of adult AD and current smoking. Both current and ever smoking were significant risk factors for adult AD, compared with non-smoking. Moreover, packs per year were significantly associated with adult AD, suggesting a lifelong cumulative risk in current smokers. In addition, non-smokers with adult AD reported significantly greater environmental tobacco exposure. Therefore, adults should be discouraged from smoking to prevent adult AD in themselves and their family members [98].

Data from German registries show that smoking AD patients have a higher disease burden with a different pattern of lesion distribution in adult AD, with a 2.5 times greater likelihood of foot involvement. Although the scoring of atopic dermatitis showed no difference between smokers and non-smokers, lesional severity of oozing, crusts, and excoriations, along with patient global assessment scores (PGA) of AD severity being higher in smokers compared to non-smokers. In addition, smokers reported increased pruritus intensity and a lower number of weeks with well-controlled AD than non-smokers. Finally, smokers had increased total IgE levels and an early age initial diagnosis of asthma. Interestingly, smokers were predominantly males (58.7%) in their forties [99]. On the other hand a study on US women, did not find a significant association between current smoking and incident AD [100].

In addition, a study of adult AD from Germany suggests increased positive screening for problematic drinking, drug use disorders, Internet addiction and gambling problems compared to the general population [101].

### 4.5. Stress

Psychological stress has long been observed to affect the course of AD. While chronic stress generally drives pathogenic immune responses, acute stress, in its effort to restore homeostasis, activates the sympathetic axis of the autonomous nervous system (SA), the endocrine hypothalamus–pituitary–adrenal axis (HPA), and the neuronal neuropeptidergic axis (NNA). This in turn causes vasoconstriction, neurogenic inflammation, and the release of pro-inflammatory neuromediators, followed by the anti-inflammatory cholinergic axis of the autonomic nervous system (CA) [102]. 

Adults with atopic dermatitis exhibit SA hyperresponsiveness, resulting in the transient release of cortisol from the adrenal glands into the bloodstream, while corticotrophin-releasing hormone (CRH), adrenocorticotropic hormone (ACTH) and cortisol from skin cells are released, which favor the Th2 response and inhibit the Th1 response. Endogenous glucocorticoids (GCs) compromise stratum corneum cohesion, epidermal barrier homeostasis and innate immunity, in normal skin [103].

Acute stress has been shown to aggravate the clinical manifestations of AD in both adults and children, and a correlation has been observed between psychosocial stress and the onset or worsening of AD. It has been shown that patients have a significantly higher sympathetic tone compared to healthy controls at rest and, in general, are less able to handle acute stress [104]. We previously mentioned the effect of the maternal psychological state during pregnancy on the development of atopic dermatitis in the offspring, but a psychological aspect of this disease exists until adulthood. Stress and sleep disturbances seem to have a two-way relationship with atopic dermatitis as both potential causes and sequelae of the disease [105,106]. The mechanisms underlying the psychological aspects of eczema across all ages involve stress responses and glucocorticoid secretion in immune dysregulation and the development of scratching behavior in response to pruritus [107,108].

## 5. Acute Stress Challenges: COVID-19 Pandemic and Russo–Ukrainian War

Despite the fact that children faced a plethora of pandemic-related issues, a reduction in air pollution and lack of contact with outdoor allergens resulted in the improvement of allergies. Preliminary data from the CORAL birth cohort revealed higher rates of egg sensitization and eczema in children born during the first pandemic lockdown [109]. Intensive hand hygiene with warm water and soap, and alcohol-based hand sanitizers (ABHS), were reported to be associated with the rapid development of hand eczema among a high proportion of young children and adults. Avoiding ABHS at school and washing their hands with a non-alcohol and additives soap and water solved their problem and brought their AD back to good control. “School triggers” seem to be important to recognize, avoid and prevent exposure [110].

During the strict government measures to contain the spread of SARS-CoV-2, intensive hand hygiene increased the risk of hand eczema, especially in AD patients (Figure 1). The use of face masks, gloves and repeated hand sanitization has been associated with high rates of adverse skin reactions among healthcare professionals with reports of acute and chronic dermatitis and secondary infection. In healthcare workers wearing protective equipment, TEWL, temperature and erythema were all significantly increased after 2 h of glove and mask use, indicating impaired epidermal barrier function. Adult AD is associated with a significant healthcare burden and loss of work productivity [111,112]. From another perspective, the COVID-19 pandemic impacted the exposome due to excessive use of disposable protective equipment, plastic packaging, and increased use of antibacterials.

The exposome is constantly being exposed to unprecedented factors, such as natural disasters or hostile activities such as the Russo–Ukrainian war in 2022 (Figure 1). There are serious long-term environmental consequences that threaten both the environment and human health. After each explosion, particles of toxic substances and heavy metals such as lead, mercury and depleted uranium are released into the air, water and soil [113]. In addition, service members encounter environmental extremes, physical stress, military gear, and hygiene difficulties, conditions which may flare an AD patient [104,114,115]. Friction, sweating and irritation can lead to increased itch and scratching, refueling the itch-scratch cycle. The real effects of natural disasters, war and pandemics will only become visible in the coming decades.

## 6. Conclusions

While genetic, epigenetic, racial diversity and epithelial barrier function could partially explain AD onset, multiple exposomal factors and acute stressors are involved across the lifespan and are equally important to understanding the development of AD. While the exposome of AD remains to be elucidated, some factors demonstrate significant age-specificity. Exposomal factors play a role starting from conception, during pregnancy, childhood, adulthood and across the lifespan with an impact on the trajectory of atopic dermatitis. Infants and young children are most affected due to PFAS, pollution and maternal nutrition. As they grow older, during adolescence, climate, infections, and rural and urban environments take their toll. Later on, lifestyle factors and stress predominate. In light of understanding the spatiotemporal effect of the exposome, investing in prevention strategies in public health and social protection is a mark of responsible action.

## References

[1] Langan S.M., Irvine A.D., Weidinger S. (2020). Atopic Dermatitis. Lancet.

[2] Moltrasio C., Romagnuolo M., Marzano A.V. (2022). Epigenetic Mechanisms of Epidermal Differentiation. Int. J. Mol. Sci..

[3] Smieszek S.P., Welsh S., Xiao C., Wang J., Polymeropoulos C., Birznieks G., Polymeropoulos M.H. (2020). Correlation of Age-of-Onset of Atopic Dermatitis with Filaggrin Loss-of-Function Variant Status. Sci. Rep..

[4] Irvine A.D., Mina-Osorio P. (2019). Disease Trajectories in Childhood Atopic Dermatitis: An Update and Practitioner’s Guide. Br. J. Dermatol..

[5] Stefanovic N., Irvine A.D., Flohr C. (2021). The Role of the Environment and Exposome in Atopic Dermatitis. Curr. Treat. Options Allergy.

[6] Ben-Gashir M.A., Seed P.T., Hay R.J. (2002). Reliance on Erythema Scores May Mask Severe Atopic Dermatitis in Black Children Compared with Their White Counterparts. Br. J. Dermatol..

[7] Mei-Yen Yong A., Tay Y.-K. (2017). Atopic Dermatitis. Dermatol. Clin..

[8] Chovatiya R., Begolka W.S., Thibau I.J., Silverberg J.I. (2021). Financial Burden and Impact of Atopic Dermatitis Out-of-Pocket Healthcare Expenses among Black Individuals in the United States. Arch. Dermatol. Res..

[9] Lusignan S., Alexander H., Broderick C., Dennis J., McGovern A., Feeney C., Flohr C. (2021). The Epidemiology of Eczema in Children and Adults in England: A Population-based Study Using Primary Care Data. Clin. Exp. Allergy.

[10] West C.E., Jenmalm M.C., Prescott S.L. (2015). The Gut Microbiota and Its Role in the Development of Allergic Disease: A Wider Perspective. Clin. Exp. Allergy.

[11] Chan C., Law B., Liu Y.-H., Ambrocio A., Au N., Jiang M., Chow K. (2018). The Association between Maternal Stress and Childhood Eczema: A Systematic Review. Int. J. Environ. Res. Public Health.

[12] Parker-Lalomio M., McCann K., Piorkowski J., Freels S., Persky V.W. (2018). Prenatal Exposure to Polychlorinated Biphenyls and Asthma, Eczema/Hay Fever, and Frequent Ear Infections. J. Asthma.

[13] Mogensen U.B., Grandjean P., Nielsen F., Weihe P., Budtz-Jørgensen E. (2015). Breastfeeding as an Exposure Pathway for Perfluorinated Alkylates. Environ. Sci. Technol..

[14] Xue J., Zartarian V., Moya J., Freeman N., Beamer P., Black K., Tulve N., Shalat S. (2007). A Meta-Analysis of Children’s Hand-to-Mouth Frequency Data for Estimating Nondietary Ingestion Exposure. Risk Anal..

[15] von Holst H., Nayak P., Dembek Z., Buehler S., Echeverria D., Fallacara D., John L. (2021). Perfluoroalkyl Substances Exposure and Immunity, Allergic Response, Infection, and Asthma in Children: Review of Epidemiologic Studies. Heliyon.

[16] Chen Q., Huang R., Hua L., Guo Y., Huang L., Zhao Y., Wang X., Zhang J. (2018). Prenatal Exposure to Perfluoroalkyl and Polyfluoroalkyl Substances and Childhood Atopic Dermatitis: A Prospective Birth Cohort Study. Environ. Health.

[17] Tsai T.-L., Wang S.-L., Hsieh C.-J., Wen H.-J., Kuo C.-C., Liu H.-J., Sun C.-W., Chen M.-L., Wu M.-T., TMICS Study Group (2021). Association between Prenatal Exposure to Metals and Atopic Dermatitis Among Children Aged 4 Years in Taiwan. JAMA Netw. Open.

[18] Just A.C., Whyatt R.M., Perzanowski M.S., Calafat A.M., Perera F.P., Goldstein I.F., Chen Q., Rundle A.G., Miller R.L. (2012). Prenatal Exposure to Butylbenzyl Phthalate and Early Eczema in an Urban Cohort. Environ. Health Perspect..

[19] Lee S., Park S.K., Park H., Lee W., Lee J.H., Hong Y.-C., Ha M., Kim Y., Lee B.-E., Ha E. (2021). Joint Association of Prenatal Bisphenol-A and Phthalates Exposure with Risk of Atopic Dermatitis in 6-Month-Old Infants. Sci. Total Environ..

[20] Shaw T.E., Currie G.P., Koudelka C.W., Simpson E.L. (2011). Eczema Prevalence in the United States: Data from the 2003 National Survey of Children’s Health. J. Investig. Dermatol..

[21] Yang S.-I., Lee S.-H., Lee S.-Y., Kim H.-C., Kim H.-B., Kim J.-H., Lim H., Park M.J., Cho H.-J., Yoon J. (2020). Prenatal PM2.5 Exposure and Vitamin D–Associated Early Persistent Atopic Dermatitis via Placental Methylation. Ann. Allergy Asthma Immunol..

[22] Foolad N., Armstrong A.W. (2014). Prebiotics and Probiotics: The Prevention and Reduction in Severity of Atopic Dermatitis in Children. Benef. Microbes.

[23] Makrgeorgou A., Leonardi-Bee J., Bath-Hextall F.J., Murrell D.F., Tang M.L., Roberts A., Boyle R.J. (2018). Probiotics for Treating Eczema. Cochrane Database Syst. Rev..

[24] Liu Y., Du X., Zhai S., Tang X., Liu C., Li W. (2022). Gut Microbiota and Atopic Dermatitis in Children: A Scoping Review. BMC Pediatr..

[25] Prescott S.L., Calder P.C. (2004). N-3 Polyunsaturated Fatty Acids and Allergic Disease. Curr. Opin. Clin. Nutr. Metab. Care.

[26] Bloomfield S.F., Rook G.A., Scott E.A., Shanahan F., Stanwell-Smith R., Turner P. (2016). Time to Abandon the Hygiene Hypothesis: New Perspectives on Allergic Disease, the Human Microbiome, Infectious Disease Prevention and the Role of Targeted Hygiene. Perspect. Public Health.

[27] Amalia N., Orchard D., Francis K.L., King E. (2020). Systematic Review and Meta-analysis on the Use of Probiotic Supplementation in Pregnant Mother, Breastfeeding Mother and Infant for the Prevention of Atopic Dermatitis in Children. Australas. J. Derm..

[28] Nicolaou N., Pancheva R., Karaglani E., Sekkidou M., Marinova-Achkar M., Popova S., Tzaki M., Kapetanaki A., Iacovidou N., Boutsikou T. (2022). The Risk Reduction Effect of a Nutritional Intervention With a Partially Hydrolyzed Whey-Based Formula on Cow’s Milk Protein Allergy and Atopic Dermatitis in High-Risk Infants Within the First 6 Months of Life: The Allergy Reduction Trial (A.R.T.), a Multicenter Double-Blinded Randomized Controlled Study. Front. Nutr..

[29] Sausenthaler S., Koletzko S., Schaaf B., Lehmann I., Borte M., Herbarth O., von Berg A., Wichmann H.-E., Heinrich J., LISA Study Group (2007). Maternal Diet during Pregnancy in Relation to Eczema and Allergic Sensitization in the Offspring at 2 y of Age. Am. J. Clin. Nutr..

[30] Gardner K.G., Gebretsadik T., Hartman T.J., Rosa M.J., Tylavsky F.A., Adgent M.A., Moore P.E., Kocak M., Bush N.R., Davis R.L. (2020). Prenatal Omega-3 and Omega-6 Polyunsaturated Fatty Acids and Childhood Atopic Dermatitis. J. Allergy Clin. Immunol. Pract..

[31] Brzozowska A., Podlecka D., Jankowska A., Król A., Kaleta D., Trafalska E., Nowakowska-Świrta E., Kałużny P., Hanke W., Bal-Gierańczyk K. (2022). Maternal Diet during Pregnancy and Risk of Allergic Diseases in Children up to 7–9 Years Old from Polish Mother and Child Cohort Study. Environ. Res..

[32] Mubanga M., Lundholm C., D’Onofrio B.M., Stratmann M., Hedman A., Almqvist C. (2021). Association of Early Life Exposure to Antibiotics With Risk of Atopic Dermatitis in Sweden. JAMA Netw. Open.

[33] Cui H., Mu Z. (2023). Prenatal Maternal Risk Factors Contributing to Atopic Dermatitis: A Systematic Review and Meta-Analysis of Cohort Studies. Ann. Derm..

[34] Silverberg J.I., Hanifin J., Simpson E.L. (2013). Climatic Factors Are Associated with Childhood Eczema Prevalence in the United States. J. Investig. Dermatol..

[35] Rueter K., Jones A.P., Siafarikas A., Chivers P., Prescott S.L., Palmer D.J. (2021). The Influence of Sunlight Exposure and Sun Protecting Behaviours on Allergic Outcomes in Early Childhood. Int. J. Environ. Res. Public Health.

[36] Camargo C.A., Ganmaa D., Sidbury R., Erdenedelger K., Radnaakhand N., Khandsuren B. (2014). Randomized Trial of Vitamin D Supplementation for Winter-Related Atopic Dermatitis in Children. J. Allergy Clin. Immunol..

[37] Thyssen J.P., Zirwas M.J., Elias P.M. (2015). Potential Role of Reduced Environmental UV Exposure as a Driver of the Current Epidemic of Atopic Dermatitis. J. Allergy Clin. Immunol..

[38] McKenzie C., Silverberg J.I. (2019). The Prevalence and Persistence of Atopic Dermatitis in Urban United States Children. Ann. Allergy Asthma Immunol..

[39] Wan J., Oganisian A., Spieker A.J., Hoffstad O.J., Mitra N., Margolis D.J., Takeshita J. (2019). Racial/Ethnic Variation in Use of Ambulatory and Emergency Care for Atopic Dermatitis among US Children. J. Investig. Dermatol..

[40] Tackett K.J., Jenkins F., Morrell D.S., McShane D.B., Burkhart C.N. (2020). Structural Racism and Its Influence on the Severity of Atopic Dermatitis in African American Children. Pediatr. Derm..

[41] Botha M., Basera W., Facey-Thomas H.E., Gaunt B., Genuneit J., Gray C.L., Kiragu W., Ramjith J., Watkins A., Levin M.E. (2019). Nutrition and Allergic Diseases in Urban and Rural Communities from the South African Food Allergy Cohort. Pediatr. Allergy Immunol..

[42] Lee J.-T., Cho Y.-S., Son J.-Y. (2010). Relationship between Ambient Ozone Concentrations and Daily Hospital Admissions for Childhood Asthma/Atopic Dermatitis in Two Cities of Korea during 2004–2005. Int. J. Environ. Health Res..

[43] Yi O., Kwon H.-J., Kim H., Ha M., Hong S.-J., Hong Y.-C., Leem J.-H., Sakong J., Lee C.G., Kim S.-Y. (2012). Effect of Environmental Tobacco Smoke on Atopic Dermatitis among Children in Korea. Environ. Res..

[44] Krämer U., Weidinger S., Darsow U., Möhrenschlager M., Ring J., Behrendt H. (2005). Seasonality in Symptom Severity Influenced by Temperature or Grass Pollen: Results of a Panel Study in Children with Eczema. J. Investig. Dermatol..

[45] Kathuria P., Silverberg J.I. (2016). Association of Pollution and Climate with Atopic Eczema in US Children. Pediatr. Allergy Immunol..

[46] Patra V., Byrne S.N., Wolf P. (2016). The Skin Microbiome: Is It Affected by UV-Induced Immune Suppression?. Front. Microbiol..

[47] Herrero-Fernandez M., Montero-Vilchez T., Diaz-Calvillo P., Romera-Vilchez M., Buendia-Eisman A., Arias-Santiago S. (2022). Impact of Water Exposure and Temperature Changes on Skin Barrier Function. J. Clin. Med..

[48] Tamagawa-Mineoka R., Katoh N. (2020). Atopic Dermatitis: Identification and Management of Complicating Factors. Int. J. Mol. Sci..

[49] Arafune J., Tsujiguchi H., Hara A., Shimizu Y., Hori D., Nguyen T.T.T., Suzuki F., Hamagishi T., Yamada Y., Nakamura H. (2021). Increased Prevalence of Atopic Dermatitis in Children Aged 0–3 Years Highly Exposed to Parabens. Int. J. Environ. Res. Public Health.

[50] Langan S.M., Silcocks P., Williams H.C. (2009). What Causes Flares of Eczema in Children?. Br. J. Dermatol..

[51] Haarala A.K., Sinikumpu S.-P., Vaaramo E., Jokelainen J., Timonen M., Auvinen J., Pekkanen J., Huilaja L. (2021). A Childhood Farm Environment Protects from Allergic Sensitization until Middle Age but Not from New-Onset Sensitization in Adulthood: A 15 Year Longitudinal Study. Int. J. Environ. Res. Public Health.

[52] Penders J., Gerhold K., Thijs C., Zimmermann K., Wahn U., Lau S., Hamelmann E. (2014). New Insights into the Hygiene Hypothesis in Allergic Diseases: Mediation of Sibling and Birth Mode Effects by the Gut Microbiota. Gut Microbes.

[53] Akdis C.A., Akdis M., Boyd S.D., Sampath V., Galli S.J., Nadeau K.C. (2023). Allergy: Mechanistic Insights into New Methods of Prevention and Therapy. Sci. Transl. Med..

[54] Herrant M., Loucoubar C., Boufkhed S., Bassène H., Sarr F.D., Baril L., Mercereau-Puijalon O., Mécheri S., Sakuntabhai A., Paul R. (2015). Risk Factors Associated with Asthma, Atopic Dermatitis and Rhinoconjunctivitis in a Rural Senegalese Cohort. Allergy Asthma Clin. Immunol..

[55] Thyssen J.P., Ahluwalia T.S., Paternoster L., Ballardini N., Bergström A., Melén E., Chawes B.L., Stokholm J., Hourihane J.O., O’Sullivan D.M. (2020). Interaction between Filaggrin Mutations and Neonatal Cat Exposure in Atopic Dermatitis. Allergy.

[56] Langan S.M., Flohr C., Williams H.C. (2007). The Role of Furry Pets in Eczema: A Systematic Review. Arch. Derm..

[57] Pelucchi C., Galeone C., Bach J.-F., La Vecchia C., Chatenoud L. (2013). Pet Exposure and Risk of Atopic Dermatitis at the Pediatric Age: A Meta-Analysis of Birth Cohort Studies. J. Allergy Clin. Immunol..

[58] Ruokolainen L., Paalanen L., Karkman A., Laatikainen T., von Hertzen L., Vlasoff T., Markelova O., Masyuk V., Auvinen P., Paulin L. (2017). Significant Disparities in Allergy Prevalence and Microbiota between the Young People in Finnish and Russian Karelia. Clin. Exp. Allergy.

[59] Haahtela T., Laatikainen T., Alenius H., Auvinen P., Fyhrquist N., Hanski I., von Hertzen L., Jousilahti P., Kosunen T.U., Markelova O. (2015). Hunt for the Origin of Allergy—Comparing the Finnish and Russian Karelia. Clin. Exp. Allergy.

[60] Wang J., Janson C., Malinovschi A., Holm M., Franklin K.A., Modig L., Johannessen A., Schlünssen V., Gislason T., Jogi N.O. (2022). Asthma, Allergic Rhinitis and Atopic Dermatitis in Association with Home Environment—The RHINE Study. Sci. Total Environ..

[61] Mahdavinia M., Greenfield L.R., Moore D., Botha M., Engen P., Gray C., Lunjani N., Hlela C., Basera W., Hobane L. (2021). House Dust Microbiota and Atopic Dermatitis; Effect of Urbanization. Pediatr. Allergy Immunol..

[62] Levin M.E., Botha M., Basera W., Facey-Thomas H.E., Gaunt B., Gray C.L., Kiragu W., Ramjith J., Watkins A., Genuneit J. (2020). Environmental Factors Associated with Allergy in Urban and Rural Children from the South African Food Allergy (SAFFA) Cohort. J. Allergy Clin. Immunol..

[63] Lunjani N., Tan G., Dreher A., Sokolowska M., Groeger D., Warwyzniak M., Altunbulakli C., Westermann P., Basera W., Hobane L. (2022). Environment-dependent Alterations of Immune Mediators in Urban and Rural South African Children with Atopic Dermatitis. Allergy.

[64] Thacher J.D., Gruzieva O., Pershagen G., Neuman Å., Wickman M., Kull I., Melén E., Bergström A. (2014). Pre- and Postnatal Exposure to Parental Smoking and Allergic Disease Through Adolescence. Pediatrics.

[65] Biagini Myers J.M., Khurana Hershey G.K. (2010). Eczema in Early Life: Genetics, the Skin Barrier, and Lessons Learned from Birth Cohort Studies. J. Pediatr..

[66] Kantor R., Kim A., Thyssen J.P., Silverberg J.I. (2016). Association of Atopic Dermatitis with Smoking: A Systematic Review and Meta-Analysis. J. Am. Acad. Dermatol..

[67] Morales E., Strachan D., Asher I., Ellwood P., Pearce N., Garcia-Marcos L. (2019). Combined Impact of Healthy Lifestyle Factors on Risk of Asthma, Rhinoconjunctivitis and Eczema in School Children: ISAAC Phase III. Thorax.

[68] Jaffary F., Faghihi G., Mokhtarian A., Hosseini S. (2015). Effects of Oral Vitamin E on Treatment of Atopic Dermatitis: A Randomized Controlled Trial. J. Res. Med. Sci..

[69] Shin J., Kim Y.J., Kwon O., Kim N.-I., Cho Y. (2016). Associations among Plasma Vitamin C, Epidermal Ceramide and Clinical Severity of Atopic Dermatitis. Nutr. Res. Pract..

[70] Oh S.-Y., Chung J., Kim M.-K., Kwon S.O., Cho B.-H. (2010). Antioxidant Nutrient Intakes and Corresponding Biomarkers Associated with the Risk of Atopic Dermatitis in Young Children. Eur. J. Clin. Nutr..

[71] Cho S.I., Lee H., Lee D.H., Kim K.-H. (2020). Association of Frequent Intake of Fast Foods, Energy Drinks, or Convenience Food with Atopic Dermatitis in Adolescents. Eur. J. Nutr..

[72] Kong H.H., Oh J., Deming C., Conlan S., Grice E.A., Beatson M.A., Nomicos E., Polley E.C., Komarow H.D., NISC Comparative Sequence Program (2012). Temporal Shifts in the Skin Microbiome Associated with Disease Flares and Treatment in Children with Atopic Dermatitis. Genome Res..

[73] Grice E.A., Kong H.H., Conlan S., Deming C.B., Davis J., Young A.C., Bouffard G.G., Blakesley R.W., Murray P.R., NISC Comparative Sequencing Program (2009). Topographical and Temporal Diversity of the Human Skin Microbiome. Science.

[74] Bjerre R.D., Holm J.B., Palleja A., Sølberg J., Skov L., Johansen J.D. (2021). Skin Dysbiosis in the Microbiome in Atopic Dermatitis Is Site-Specific and Involves Bacteria, Fungus and Virus. BMC Microbiol..

[75] Bisgaard H., Li N., Bonnelykke K., Chawes B.L.K., Skov T., Paludan-Müller G., Stokholm J., Smith B., Krogfelt K.A. (2011). Reduced Diversity of the Intestinal Microbiota during Infancy Is Associated with Increased Risk of Allergic Disease at School Age. J. Allergy Clin. Immunol..

[76] Namara B., Nash S., Lule S.A., Akurut H., Mpairwe H., Akello F., Tumusiime J., Kizza M., Kabagenyi J., Nkurunungi G. (2017). Effects of Treating Helminths during Pregnancy and Early Childhood on Risk of Allergy-Related Outcomes: Follow-up of a Randomized Controlled Trial. Pediatr. Allergy Immunol..

[77] Lee H.H., Patel K.R., Singam V., Rastogi S., Silverberg J.I. (2019). A Systematic Review and Meta-Analysis of the Prevalence and Phenotype of Adult-Onset Atopic Dermatitis. J. Am. Acad. Dermatol..

[78] Niwa Y., Sumi H., Kawahira K., Terashima T., Nakamura T., Akamatsu H. (2003). Protein Oxidative Damage in the Stratum Corneum: Evidence for a Link between Environmental Oxidants and the Changing Prevalence and Nature of Atopic Dermatitis in Japan. Br. J. Derm..

[79] Folster-Holst R., Pape M., Buss Y.L., Christophers E., Weichenthal M. (2006). Low Prevalence of the Intrinsic Form of Atopic Dermatitis among Adult Patients. Allergy.

[80] Tang K.-T., Ku K.-C., Chen D.-Y., Lin C.-H., Tsuang B.-J., Chen Y.-H. (2017). Adult Atopic Dermatitis and Exposure to Air Pollutants—A Nationwide Population-Based Study. Ann. Allergy Asthma Immunol..

[81] Kantor R., Silverberg J.I. (2017). Environmental Risk Factors and Their Role in the Management of Atopic Dermatitis. Expert Rev. Clin. Immunol..

[82] Rothenberg M.E. (2022). The Climate Change Hypothesis for the Allergy Epidemic. J. Allergy Clin. Immunol..

[83] Kim K. (2015). Influences of Environmental Chemicals on Atopic Dermatitis. Toxicol. Res..

[84] Tang K.-T., Chen P.-A., Lee M.-R., Lee M.-F., Chen Y.-H. (2020). The Relationship between Exposure to Polycyclic Aromatic Hydrocarbons and Adult Atopic Dermatitis. Asian Pac. J. Allergy Immunol..

[85] Hidaka T., Ogawa E., Kobayashi E.H., Suzuki T., Funayama R., Nagashima T., Fujimura T., Aiba S., Nakayama K., Okuyama R. (2017). The Aryl Hydrocarbon Receptor AhR Links Atopic Dermatitis and Air Pollution via Induction of the Neurotrophic Factor Artemin. Nat. Immunol..

[86] Hendricks A.J., Eichenfield L.F., Shi V.Y. (2020). The Impact of Airborne Pollution on Atopic Dermatitis: A Literature Review. Br. J. Derm..

[87] Kabashima K., Otsuka A., Nomura T. (2017). Linking Air Pollution to Atopic Dermatitis. Nat. Immunol..

[88] Wesley N.O., Maibach H.I. (2003). Racial (Ethnic) Differences in Skin Properties: The Objective Data. Am. J. Clin. Dermatol..

[89] Pesce G., Marcon A., Carosso A., Antonicelli L., Cazzoletti L., Ferrari M., Fois A.G., Marchetti P., Olivieri M., Pirina P. (2015). Adult Eczema in Italy: Prevalence and Associations with Environmental Factors. J. Eur. Acad. Derm. Venereol..

[90] Nnoruka E.N. (2004). Current Epidemiology of Atopic Dermatitis in South-Eastern Nigeria. Int. J. Derm..

[91] Silverberg J.I., Hanifin J.M. (2013). Adult Eczema Prevalence and Associations with Asthma and Other Health and Demographic Factors: A US Population–Based Study. J. Allergy Clin. Immunol..

[92] Balić A., Vlašić D., Žužul K., Marinović B., Bukvić Mokos Z. (2020). Omega-3 Versus Omega-6 Polyunsaturated Fatty Acids in the Prevention and Treatment of Inflammatory Skin Diseases. Int. J. Mol. Sci..

[93] Eriksen B.B., Kåre D.L. (2006). Open Trial of Supplements of Omega 3 and 6 Fatty Acids, Vitamins and Minerals in Atopic Dermatitis. J. Dermatol. Treat..

[94] Ito M., Morita T., Okazaki S., Koto M., Ichikawa Y., Takayama R., Hoashi T., Saeki H., Kanda N. (2019). Dietary Habits in Adult Japanese Patients with Atopic Dermatitis. J. Derm..

[95] Alfonso F., Goicolea J., Hernández R., Bañuelos C., Segovia J., Fernández-Ortiz A., Gonçalves M., Alonso L., Macaya C. (1995). [Coronary angioscopy: Initial experience during coronary interventions]. Rev. Esp. Cardiol..

[96] Park S., Bae J.-H. (2016). Fermented Food Intake Is Associated with a Reduced Likelihood of Atopic Dermatitis in an Adult Population (Korean National Health and Nutrition Examination Survey 2012-2013). Nutr. Res..

[97] Silverberg J.I., Greenland P. (2015). Eczema and Cardiovascular Risk Factors in 2 US Adult Population Studies. J. Allergy Clin. Immunol..

[98] Lee C.H., Chuang H.Y., Hong C.H., Huang S.K., Chang Y.C., Ko Y.C., Yu H.S. (2011). Lifetime Exposure to Cigarette Smoking and the Development of Adult-Onset Atopic Dermatitis: Smoking and Adult Atopic Dermatitis. Br. J. Dermatol..

[99] Pilz A.C., Schielein M.C., Schuster B., Heinrich L., Haufe E., Abraham S., Heratizadeh A., Harder I., Kleinheinz A., Wollenberg A. (2022). Atopic Dermatitis: Disease Characteristics and Comorbidities in Smoking and Non-smoking Patients from the TREATgermany Registry. Acad. Derm. Venereol..

[100] Morra D.E., Cho E., Li T., Camargo C.A., Qureshi A.A., Drucker A.M. (2021). Smoking and Risk of Adult-Onset Atopic Dermatitis in US Women. J. Am. Acad. Dermatol..

[101] Pilz A.C., Durner V., Schielein M.C., Schuster B., Beckmann J., Biedermann T., Eyerich K., Zink A. (2022). Addictions in Patients with Atopic Dermatitis: A Cross-sectional Pilot Study in Germany. J. Eur. Acad. Derm. Venereol..

[102] Pondeljak N., Lugović-Mihić L. (2020). Stress-Induced Interaction of Skin Immune Cells, Hormones, and Neurotransmitters. Clin. Ther..

[103] Seiffert K., Hilbert E., Schaechinger H., Zouboulis C.C., Deter H.-C. (2005). Psychophysiological Reactivity under Mental Stress in Atopic Dermatitis. Dermatology.

[104] Kodama A., Horikawa T., Suzuki T., Ajiki W., Takashima T., Harada S., Ichihashi M. (1999). Effect of Stress on Atopic Dermatitis: Investigation in Patients after the Great Hanshin Earthquake. J. Allergy Clin. Immunol..

[105] Sanders K.M., Akiyama T. (2018). The Vicious Cycle of Itch and Anxiety. Neurosci. Biobehav. Rev..

[106] Silverberg J.I., Garg N.K., Paller A.S., Fishbein A.B., Zee P.C. (2015). Sleep Disturbances in Adults with Eczema Are Associated with Impaired Overall Health: A US Population-Based Study. J. Investig. Dermatol..

[107] Steinhoff M., Suárez A., Feramisco J., Koo J. (2012). Psychoneuroimmunology of Psychological Stress and Atopic Dermatitis: Pathophysiologic and Therapeutic Updates. Acta Derm. Venerol..

[108] Yosipovitch G., Tran B., Papoiu A., Russoniello C., Wang H., Patel T., Chan Y. (2010). Effect of Itch, Scratching and Mental Stress on Autonomic Nervous System Function in Atopic Dermatitis. Acta Derm. Venerol..

[109] Hurley S., Franklin R., McCallion N., Byrne A.M., Fitzsimons J., Byrne S., White M., O’Mahony L., Hourihane J.O. (2022). Allergy-related Outcomes at 12 Months in the CORAL Birth Cohort of Irish Children Born during the First COVID 19 Lockdown. Pediatr. Allergy Immunol..

[110] Singh M., Pawar M., Bothra A., Choudhary N. (2020). Overzealous Hand Hygiene during the COVID 19 Pandemic Causing an Increased Incidence of Hand Eczema among General Population. J. Am. Acad. Dermatol..

[111] Pecoraro L., Chiaffoni G., Piacentini G., Pietrobelli A. (2022). The Need of an Updated Culture of “Occupational” Atopic Hand Dermatitis in Children at the Time of COVID-19. Acta Biomed..

[112] Rundle C.W., Presley C.L., Militello M., Barber C., Powell D.L., Jacob S.E., Atwater A.R., Watsky K.L., Yu J., Dunnick C.A. (2020). Hand Hygiene during COVID-19: Recommendations from the American Contact Dermatitis Society. J. Am. Acad. Dermatol..

[113] Pereira P., Bašić F., Bogunovic I., Barcelo D. (2022). Russian-Ukrainian War Impacts the Total Environment. Sci. Total Environ..

[114] Riegleman K.L., Farnsworth G.S., Wong E.B. (2019). Atopic Dermatitis in the US Military. Cutis.

[115] (2022). The Lancet Ukraine’s Humanitarian Disaster: Priorities for Health. Lancet.

